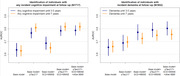# Combining blood‐based biomarkers and accessible measures for the prognosis of cognitive decline in older primary care patients

**DOI:** 10.1002/alz.092239

**Published:** 2025-01-09

**Authors:** Luca Kleineidam, Selçuk Oezdemir, Pamela Martino‐Adami, Madhurima Chatterjee, Michael Pentzek, Siegfried Weyerer, Horst Bickel, Birgitt Wiese, Steffi G. Riedel‐Heller, Martin Scherer, Henrik Zetterberg, Kaj Blennow, Andreas Jeromin, Michael Wagner, Alfredo Ramirez, Anja Schneider

**Affiliations:** ^1^ Department of Neurodegenerative Diseases and Geriatric Psychiatry, University of Bonn Medical Center, Bonn Germany; ^2^ German Center for Neurodegenerative Diseases (DZNE), Bonn Germany; ^3^ Ataturk University, Department of Genetics, Erzurum Turkey; ^4^ Division of Neurogenetics and Molecular Psychiatry, Department of Psychiatry and Psychotherapy, Faculty of Medicine and University Hospital Cologne, University of Cologne, Cologne Germany; ^5^ University Duisburg‐Essen, Medical Faculty, Institute of General Practice, Primary Care Research, Essen, NRW Germany; ^6^ Central Institute of Mental Health, Medical Faculty Mannheim, Heidelberg University, Mannheim, Germany, Mannheim Germany; ^7^ Department of Psychiatry, Technical University, Munich, Germany, Munich Germany; ^8^ Institute for General Practice, Hannover Medical School, Hannover, Lower Saxony Germany; ^9^ Institute of Social Medicine, Occupational Health and Public Health (ISAP), Medical Faculty, University of Leipzig, Leipzig Germany; ^10^ Department of Primary Medical Care, University Medical Center Hamburg, Hamburg Germany; ^11^ Department of Neurodegenerative Disease, UCL Institute of Neurology, London United Kingdom; ^12^ UK Dementia Research Institute, University College London, London United Kingdom; ^13^ Clinical Neurochemistry Laboratory, Sahlgrenska University Hospital, Mölndal Sweden; ^14^ Hong Kong Center for Neurodegenerative Diseases, Clear Water Bay Hong Kong; ^15^ Department of Psychiatry and Neurochemistry, Institute of Neuroscience and Physiology, The Sahlgrenska Academy, University of Gothenburg, Mölndal, Gothenburg Sweden; ^16^ Department of Psychiatry and Neurochemistry, Institute of Neuroscience and Physiology, The Sahlgrenska Academy, University of Gothenburg, Mölndal Sweden; ^17^ ALZpath. Inc, Carlsbad, CA USA; ^18^ Cluster of Excellence Cellular Stress Responses in Aging‐Associated Diseases (CECAD), University of Cologne, 50931, Cologne Germany; ^19^ Department of Psychiatry and Glenn, Biggs Institute for Alzheimer’s and Neurodegenerative Diseases, San Antonio, TX USA

## Abstract

**Background:**

Combining plasma phosphorylated Tau 217 (pTau217), with cognitive assessments allows for predicting incident dementia in mild cognitive impairment (MCI). However, the performance of this approach in primary care as well as the added value of other blood‐based biomarkers (BBM) in this setting is unclear.

**Methods:**

We examined 833 older primary care patients from the AgeCoDe/AgeQualiDe study (median age=83). Plasma ALZpath pTau217 and other blood‐based biomarkers (BBM; pTau181, GFAP, NfL, Abeta‐42/40) were measured using single‐molecule arrays. Subjective cognitive decline (SCD) and the Mini‐Mental State Examination (MMSE) were assessed at baseline. We evaluated the performance of BBM, MMSE, and SCD reports for predicting the development of any cognitive impairment (MCI or dementia) or conversion to dementia at 3.5 and 7 years of follow‐up using time‐dependent ROC analysis with death as competing risk.

**Results:**

Plasma pTau217 improved the prognosis of developing any cognitive impairment at follow‐up beyond age, sex, and renal function (base model; Figure 1) and showed a high prognostic value at 7 years of follow‐up (p<0.001, AUC=0.809; 3.5 years prognosis: p<0.001; AUC=0.767). Adding SCD and MMSE to the model provided only a small improvement in short‐term prediction (3.5 years prognosis: p=0.002; AUC=0.785) while no significant improvements were observed by considering other BBM.

For predicting incident dementia, pTau217 also increased the prognostic value beyond the base model (3.5 years: p=0.044, AUC=0.689; 7 years: p=0.002; AUC=0.697). However, this increase was smaller as compared to the prognosis of any cognitive impairment since individuals developing MCI but not dementia showed similar pTau217 levels at baseline. Furthermore, considering MMSE and SCD reports resulted in a considerable increase in prognostic value beyond pTau217 (3.5 years: p<0.001, AUC=0.791; 7 years: p=0.002; AUC=0.749). Plasma pTau217 in turn improved the long‐term prediction beyond SCD reports and MMSE (p<0.001, AUC=0.749 vs 0.724). Again, other BBM did not increase the prognostic value.

**Conclusions:**

In our sample, pTau217 showed a high ability to discriminate between older primary care patients who will stay cognitively stable over seven years from patients developing any cognitive impairment, in contrast to other BBM. Assessments of SCD and cognition can further improve the identification of patients at increased short‐term risk for dementia.